# Bayesian analysis of generalized log-Burr family with R

**DOI:** 10.1186/2193-1801-3-185

**Published:** 2014-04-10

**Authors:** Md Tanwir Akhtar, Athar Ali Khan

**Affiliations:** Department of Statistics and Operations Research, Aligarh Muslim University, Aligarh, 202002 India

**Keywords:** Bayesian, Log-Burr, Laplace approximation, Simulation, Laplace’s Demon, Posterior density

## Abstract

Log-Burr distribution is a generalization of logistic and extreme value distributions, which are important reliability models. In this paper, Bayesian approach is used to model reliability data for log-Burr model using analytic and simulation tools. Laplace approximation is implemented for approximating posterior densities of the parameters. Moreover, parallel simulation tools are also implemented using ‘LaplacesDemon’ package of R.

## Introduction

The log-Burr distribution is a generalization of two important reliability models, that is, logistic distribution and extreme value distribution. The non-Bayesian analysis of generalized log-Burr distribution is a very difficult task, whereas it can be a routine analysis when dealing in a Bayesian paradigm. In this paper, an attempt has been made with the following objectives:

- To define a Bayesian model, that is, specification of likelihood and prior distribution.
- To write down the R code for approximating posterior densities with Laplace approximation and simulation tools (R Core Team, 2013).
- To illustrate numeric as well as graphic summaries of the posterior densities.

## The log location-scale model

The probability density function of a parametric location-scale model for a random variable *y* on (−*∞*,*∞*) with location parameter *μ* (−*∞*<*μ*<*∞*) and scale parameter *σ* (>0) is given by1

The corresponding distribution and reliability function for *y* are

The standardized random variable *z*=(*y*−*μ*)/*σ* clearly has density and reliability functions *f*_0_(*z*) and *R*_0_(*z*) respectively, and Equation (1) with *μ*=0 and *σ*=1 is called the standard form of the distribution.

The lifetime distribution, that is, exponential, Weibull, all have the property that *y*=*logt* has a location scale distribution: the Weibull, log-normal, and log-logistic distribution for *t* correspond to extreme value, normal, and logistic distributions for y. The reliability functions for *z*=(*y*−*μ*)/*σ* on (−*∞*,*∞*) are respectively,

Similarly, any location-scale model (Equation (1)) gives a lifetime distribution by the transformation *t*=exp(*y*). In this case the reliability function can be expressed as

where *α*=exp(*μ*), *β*=1/*σ* and  is a reliability function defined on (0,*∞*) (e.g., Lawless 2003).

The log-Burr distribution can be obtained by generalizing a parametric location-scale family of distribution given by Equation (1), to let pdf, cdf, or reliability function include one or more parameters. This distribution is much useful because they include common two parameter lifetime distributions as special cases.

## The generalized log-Burr family

**Figure 1:**
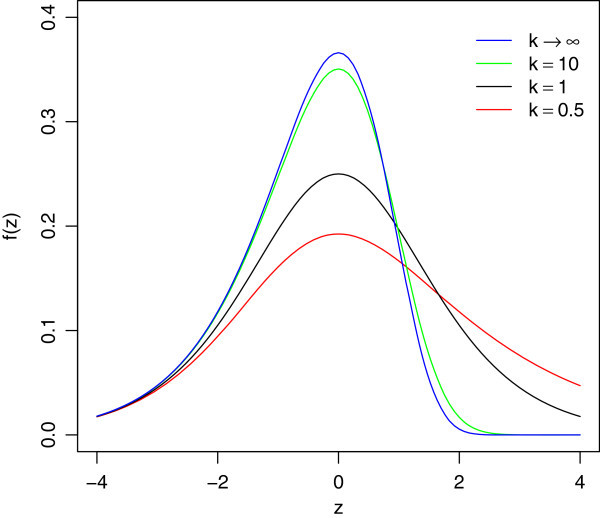
**Probability density function of log-Burr distribution for** ***k*** **=0** ***.*** **5,1,10,** ***∞*** **.**

The generalized log-Burr family, for which the standardized variable *z*=(*y*−*μ*)/*σ* has the probability density function of the form

and the corresponding reliability function

where *k* (>0) is a shape parameter. The special case, *k*=1 gives the logistic distribution and *k*→*∞* gives the extreme value distribution. Since the generalized log-Burr family includes log-logistic and Weibull distributions, it allows discrimination between them. It is also a flexible model for fitting the lifetime data (e.g., Lawless 2003). Figure 1 shows probability density functions for log-Burr distributions with different values of *k*.

## The half-Cauchy prior distribution

The probability density function of half-Cauchy distribution with scale parameter *α* is given by

**Figure 2:**
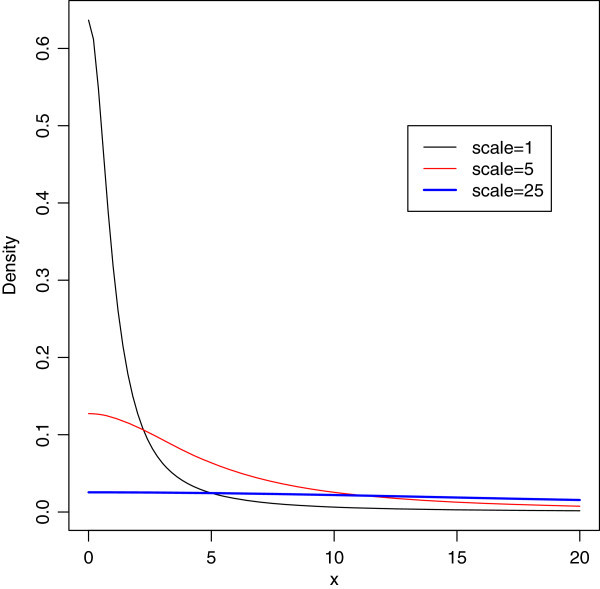
**It is evident from the above plot that for scale = 25 the half-Cauchy distribution becomes almost uniform.**

The mean and variance of the half-Cauchy distribution do not exist, but its mode is equal to 0. The half-Cauchy distribution with scale *α*=25 is a recommended, default, noninformative prior distribution for a scale parameter. At this scale *α*=25, the density of half-Cauchy is nearly flat but not completely (see Figure 2), prior distributions that are not completely flat provide enough information for the numerical approximation algorithm to continue to explore the target density, the posterior distribution. The inverse-gamma is often used as a noninformative prior distribution for scale parameter, however, this model creates problem for scale parameters near zero, Gelman and Hill (2007) recommend that, the uniform, or if more information is necessary the half-Cauchy is a better choice. Thus, in this paper, the half-Cauchy distribution with scale parameter *α*=25 is used as a noninformative prior distribution.

## The Laplace approximation

Many simple Bayesian analyses based on noninformative prior distribution give similar results to standard non-Bayesian approaches, for example, the posterior *t*-interval for the normal mean with unknown variance. The extent to which a noninformative prior distribution can be justified as an objective assumption depends on the amount of information available in the data; in the simple cases as the sample size *n* increases, the influence of the prior distribution on posterior inference decreases. These ideas, sometime referred to as asymptotic approximation theory because they refer to properties that hold in the limit as *n* becomes large. Thus, a remarkable method of asymptotic approximation is the Laplace approximation which accurately approximates the unimodal posterior moments and marginal posterior densities in many cases. In this section we introduce a brief, informal description of Laplace approximation method.

Suppose −*h*(*θ*) is a smooth, bounded unimodal function, with a maximum at , and *θ* is a scalar. By Laplace’s method (e.g., Tierney and Kadane 1986), the integral

can be approximated by

where

As presented in Mosteller and Wallace (1964), Laplace’s method is to expand about  to obtain:

Recalling that , we have

Intuitively, if exp[ −*nh*(*θ*)] is very peaked about , then the integral can be well approximated by the behavior of the integrand near . More formally, it can be shown that

To calculate moments of posterior distributions, we need to evaluate expressions such as:2

where exp[ −*nh*(*θ*)]=*L*(*θ*|*y*)*p*(*θ*) (e.g., Tanner 1996).

## Fitting of intercept model

### Fitting with LaplaceApproxomation

The Laplace approximation is a family of asymptotic techniques used to approximate integrals (Statisticat LLC 2013). It seems to accurately approximate uni-modal posterior moments and marginal posterior densities in many cases. Here, for fitting of linear regression model we use the function LaplaceApproximation which is an implementation of Laplace’s approximations of the integrals involved in the Bayesian analysis of the parameters in the modeling process. This function deterministically maximizes the logarithm of the unnormalized joint posterior density using one of the several optimization techniques. The aim of Laplace approximation is to estimate posterior mode and variance of each parameter. For getting posterior modes of the log-posteriors, a number of optimization algorithms are implemented. This includes Levenberg-Marquardt (LM) algorithm which is default. However, we find that the Limited-Memory BFGS (L-BFGS) is a better alternative in Bayesian scenario. The limited-memory BFGS (Broyden-Fletcher-Goldfarb-Shanno) algorithm is a quasi-Newton optimization algorithm that compactly approximates the Hessian matrix. Rather than storing the dense Hessian matrix, L-BFGS stores only a few vectors that represent the approximation. It may be noted that Newton-Raphson is the last choice as it is very sensitive to the starting values, it creates problems when starting values are far from the targets, and calculating and inverting the Hessian matrix can be computationally expensive, although it is also implemented in LaplaceApproximation for the sake of completion. The main arguments of LaplaceApproximation can be seen by using the function args as

First argument Model defines the model to be implemented, which contains specification of likelihood and prior. LaplaceApproximation passes two argument to the model function, parm and Data, and receives five arguments from the model function: LP (the logarithm of the unnormalized joined posterior density), Dev (the deviance), Monitor (the monitored variables), yhat (the variables for the posterior predictive checks), and parm, the vector of parameters, which may be constrained in the model function. The argument parm requires a vector of initial values equal in length to the number of parameters, and LaplaceApproximation will attempt to optimize these initial values for the parameters, where the optimized values are the posterior modes. The Data argument requires a listed data which must be include variable names and parameter names. The argument sir=TRUE stands for implementation of sampling importance resampling algorithm, which is a bootstrap procedure to draw independent sample with replacement from the posterior sample with unequal sampling probabilities. Contrary to sir of LearnBayes package, here proposal density is multivariate normal and not t.

#### Locomotive controls data

Let us introduce a failure times dataset taken from Lawless (2003), so that all the concepts and computations will be discussed around that data. The same data were discussed by Schmee and Nelson (1977). This data set contains the number of thousand miles at which different locomotive controls failed, in a life test involving 96 controls. The test was terminated after 135,000 miles, by which time 37 failures had occurred. The failure times for the 37 failed units are 22.5, 37.5, 46.0, 48.5, 51.5, 53.0, 54.5, 57.5, 66.5, 68.0, 69.5, 76.5, 77.0, 78.5, 80.0, 81.5, 82.0, 83.0, 84.0, 91.5, 93.5, 102.5, 107.0, 108.5, 112.5, 113.5, 116.0, 117.0, 118.5, 119.0, 120.0, 122.5, 123.0, 127.5, 131.0, 132.5, 134.0. In addition, there are 59 censoring times, all equal to 135.0.

#### Creation of data

The function LaplaceApproximation requires data that is specified in a list. For *locomotive controls data* the logarithm of failTime will be the response variable. Since intercept is the only term in the model, a vector of 1’s is inserted into designed matrix *X*. Thus, *J* = 1 indicates only column of 1’s in the matrix.

In this case, there are two parameters beta and log.sigma which must be specified in vector parm.names. The logposterior LP and sigma are included as monitored variables in vector mon.names. The number of observations are specified by *N*. Censoring is also taken into account, where 0 stands for censored and 1 for uncensored values. Finally all these thing are combined in a listed form as MyData object at the end of the command.

#### Initial values

The function LaplaceApproximation requires a vector of initial values for the parameters. Each initial value is a starting point for the estimation of a parameter. Here, the first parameter, the beta has been set equal to zero, and the remaining parameter, log.sigma, has been set equal to log(1), which is zero. The order of the elements of the initial values must match the order of the parameters. Thus, define a vector of initial values

For initial values the function GIV (which stands for “Generate Initial Values”) may also be used to randomly generate initial values.

#### Model specification

The function LaplaceApproximation can fit any Bayesian model for which likelihood and prior are specified. However, it is equally useful for maximum likelihood estimation. To use this method one must specify a model. Thus, for fitting of the locomotive controls data, consider that the logarithm of failTime follows log-Burr distribution which is often written as

and expectation vector *μ* is equal to the inner product of design matrix *X* and parameter *β*

Prior probabilities are specified for regression coefficient *β* and scale parameter *σ*

The large variance and small precision indicate a lot of uncertainty of each *β*, and is hence a weakly informative prior distribution. Similarly, half-Cauchy is a weakly informative prior for *σ*.

The Model function contains two arguments, that is, parm and Data, where parm is for the set of parameters, and Data is the list of data. There are two parameters beta and sigma having priors beta.prior and sigma.prior, respectively. The object LL stands for loglikelihood and LP stands for logposterior. The function Model returns the object Modelout, which contains five objects in listed form that includes logposterior LP, deviance Dev, monitoring parameters Monitor, fitted values yhat and estimates of parameters parm.

#### Model fitting

To fit the above specified model, the function LaplaceApproximation is used and its results are assigned to object Fit. Its summary of results are printed by the function print, which prints detailed summary of results and it is not possible to show here. However, its relevant parts are summarized in the next section.

#### Summarizing output

The function LaplaceApproximation approximates the posterior density of the fitted model, and posterior summaries can be seen in the following tables. Table 1 represents the analytic result using Laplace approximation method while Table 2 represents the simulated results using sampling importance resampling algorithm. From these posterior summaries, it is obvious that, the posterior mode of intercept parameter *β*_0_ for logistic distribution is 5.08±0.09 whereas posterior mode of log(*σ*)b is −0.96±0.15, while for Weibull distribution the posterior mode of intercept parameter *β*_0_ is 5.21±0.09 whereas posterior mode of log(*σ*) is −0.85±0.15. Both the parameters of different distributions are statistically significant also. In a practical data analysis, intercept model is discussed merely as a beginning point. More meaningful model is simple regression model or multiple regression model, which will be discussed in Section ‘Fitting of regression model’. Simulation tools are being discussed in the next section.

**Table 1:** **Summary of the analytic approximation using the function** LaplaceApproximation. It may be noted that these summaries are based on asymptotic approximation, and hence Mode stands for posterior mode, SD stands for posterior standard deviation, and LB, UB **are 2.5% and 97.5% quantiles, respectively**

Logistic model (k =1)				
**Parameter**	**Mode**	**SD**	**LB**	**UB**
Beta	5.08	0.09	4.90	5.26
Log.sigma	-0.96	0.15	-1.25	-0.66
**Weibull model (k =30)**				
**Parameter**	**Mode**	**SD**	**LB**	**UB**
Beta	5.21	0.09	5.03	5.39
Log.sigma	-0.85	0.15	-1.16	-0.54

**Table 2:** **Summary matrices of the simulation due to sampling importance resampling algorithm using the function** LaplaceApproximation **, where** Mean **stands for posterior mean,** SD **for posterior standard deviation,** MCSE **for Monte Carlo standard error,** ESS **, for effective sample size, and** LB **,** Median **,** UB **are 2.5%, 50%, 97.5% quantiles, respectively**

Logistic model (k =1)							
**Parameter**	**Mean**	**SD**	**MCSE**	**ESS**	**LB**	**Median**	**UB**
Beta	5.09	0.09	0.00	1000	4.93	5.09	5.27
Log.sigma	-0.93	0.14	0.00	1000	-1.22	-0.93	-0.65
Deviance	149.04	1.81	0.06	1000	147.24	148.45	153.94
LP	-86.02	0.90	0.03	1000	-88.47	-85.72	-85.12
Sigma	0.40	0.06	0.00	1000	0.29	0.39	0.52
**Weibull model (k=30)**							
**Parameter**	**Mean**	**SD**	**MCSE**	**ESS**	**LB**	**Median**	**UB**
Beta	5.22	0.09	0.00	1000	5.06	5.21	5.40
Log.sigma	-0.82	0.15	0.00	1000	-1.10	-0.82	-0.51
Deviance	149.44	1.94	0.06	1000	147.52	148.87	154.61
LP	-86.22	0.97	0.03	1000	-88.80	-85.93	-85.26
Sigma	0.45	0.07	0.00	1000	0.33	0.44	0.60

### Fitting with LaplacesDemon

Now we have to analyze the same data with the function LaplacesDemon, which is the main function of Laplace’s Demon. Given data, a model specification, and initial values, LaplacesDemon maximizes the logarithm of the unnormalized joint posterior density with Markov chain Monte Carlo (MCMC) algorithms, also called samplers, and provides samples of the marginal posterior distributions, deviance and other monitored variables. Laplace’s Demon offers a large number of MCMC algorithms for numerical approximation. Popular families include Gibbs sampling, Metropolis-Hasting (MH), Random-Walk-Metropolis (RWM), slice sampling, Metropolis-within Gibbs (MWG), Adaptive-Metropolis-within-Gibbs (AMWG), and many others. However, details of MCMC algorithms are best explored online at http://www.bayesian-inference.com/mcmc, as well as in the “LaplacesDemon Tutorial" vignette. The main arguments of the LaplacesDemon can be seen by using the function args as:

The arguments Model and Data specify the model to be implemented and list of data, which are need not to define here for the function LaplacesDemon as they are already defined for LaplaceApproximation. Initial.Values requires a vector of initial values equal in length to the number of parameter. The argument Covar= NULL indicates that variance vector or covariance matrix has not been specified, so the algorithm will begin with its own estimates. Next two arguments Iterations= 100000 and Status= 1000 indicates that the LaplacesDemon function will update 10000 times before completion and status is reported after every 1000 iterations. The thinning argument accepts integers between 1 and number of iterations, and indicates that every 100th iteration will be retained, while the others are discarded. Thinning is performed to reduced autocorrelation and the number of marginal posterior samples. Further, the Algorithm requires abbreviated name of the MCMC algorithm in quotes. In this case RWM is short for the Random-Walk-Metropolis. Finally, Specs= Null is default argument, and accepts a list of specifications for the MCMC algorithm declared in the Algorithm argument.

#### Initial values

Laplace’s Demon requires a vector of initial values for the parameters. Each initial value will be the starting point for an adaptive chain, or a non-adaptive Markov chain of a parameter. If all initial values are set to zero, then Laplace’s Demon will attempt to optimize the initial values with the LaplaceApproximation function using a resilent backpropagation algorithm. So, it is better to use the last fitted object Fit with the function as.initial.values to get a vector of initial values from the LaplaceApproximation for fitting of LaplacesDemon. Thus, to obtain a vector of initial values the function as.initial.values is used as

#### Model fitting

Laplace’s Demon is stochastic, or involves pseudo-random numbers, its better to set a seed with set.seed function for pseudo-random number generation before fitting with LaplacesDemon, so results can be reproduced. Now, fit the prespecified model with the function LaplacesDemon, and its results are assigned to the object name FitDemon. Its summary of results are printed with the function print, and its relevant parts are summarized in the next section.

#### Summarizing output

**Figure 3:**
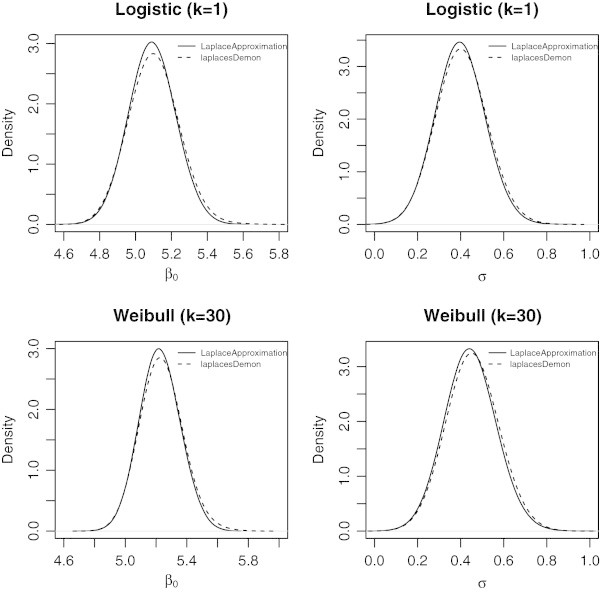
**Plot of posterior densities of the parameters** ***β***_**0**_ **and** ***σ*** **for the posterior distribution of log-Burr model using the functions** LaplaceApproximation and LaplacesDemon **.** It is evident from these plots that LaplceApproximation is excellent as it resembles with LaplacesDemon. The difference between the two seems magical.

The LaplacesDemon simulates the data from the posterior density with *Random-Walk Metropolis* and approximate the results which can be seen in the in the following tables. Table 3 represents the simulated results in a matrix form that summarizes the marginal posterior distributions of the parameters over all samples which contains mean, standard deviation, MCSE (Monte Carlo Standard Error), ESS (Effective Sample Size), and finally 2.5*%*, 50%, 97.5*%* quantiles, and Table 4 summarizes the simulated results due to stationary samples. The complete picture of the results can also be seen in Figure 3.

**Table 3:** **Posterior summaries of simulation due to all samples using the function** LaplacesDemon

Logistic model (k =1)							
**Parameter**	**Mean**	**SD**	**MCSE**	**ESS**	**LB**	**Median**	**UB**
Beta	5.10	0.10	0.01	481.56	4.92	5.10	5.30
Log.sigma	-0.92	0.16	0.01	427.68	-1.25	-0.91	-0.59
Deviance	149.55	2.31	0.18	360.81	147.27	149.05	155.11
LP	-86.27	1.15	0.09	360.82	-89.05	-86.02	-85.13
Sigma	0.40	0.07	0.00	442.40	0.29	0.40	0.55
**Weibull model (k =30)**							
**Parameter**	**Mean**	**SD**	**MCSE**	**ESS**	**LB**	**Median**	**UB**
Beta	5.24	0.10	0.01	373.50	5.07	5.22	5.46
Log.sigma	-0.80	0.16	0.01	360.03	-1.11	-0.79	-0.50
Deviance	149.62	2.14	0.15	334.67	147.55	148.94	155.09
LP	-86.31	1.07	0.07	334.66	-89.04	-85.97	-85.27
Sigma	0.46	0.07	0.00	373.10	0.33	0.45	0.61

**Table 4:** **Posterior summaries of simulation due to stationary samples using the function** LaplacesDemon

Logistic model (k =1)							
**Parameter**	**Mean**	**SD**	**MCSE**	**ESS**	**LB**	**Median**	**UB**
Beta	5.10	0.10	0.01	481.56	4.92	5.10	5.30
Log.sigma	-0.92	0.16	0.01	427.68	-1.25	-0.91	-0.59
Deviance	149.55	2.31	0.18	360.81	147.27	149.05	155.11
LP	-86.27	1.15	0.09	360.82	-89.05	-86.02	-85.13
Sigma	0.40	0.07	0.00	442.40	0.29	0.40	0.55
**Weibull model (k =30)**							
**Parameter**	**Mean**	**SD**	**MCSE**	**ESS**	**LB**	**Median**	**UB**
Beta	5.24	0.10	0.01	373.50	5.07	5.22	5.46
Log.sigma	-0.80	0.16	0.01	360.03	-1.11	-0.79	-0.50
Deviance	149.62	2.14	0.15	334.67	147.55	148.94	155.09
LP	-86.31	1.07	0.07	334.66	-89.04	-85.97	-85.27
Sigma	0.46	0.07	0.00	373.10	0.33	0.45	0.61

## Fitting of regression model

### Fitting with LaplaceApproxomation

#### Electrical insulating fluid failure times data

Let us introduce a failure times data set of electrical insulating fluid for fitting of regression model, which is taken from Lawless (2003). The same data set is discussed in Nelson (1972). Nelson (1972) described the results of a life test experiment in which specimen of a type of electrical insulating fluid were subjected to a constant voltage stress. The length of time until each specimen failed, or “broke down” was observed. The data give results for seven groups of specimen, tested as voltage ranging from 26 to 38 kilovolts (kV).

#### Data creation

For fitting of failure times of electrical insulating fluid data with LaplaceApproximation, the logarithm of breakdownTime will be the response variable and voltageLevel will be the regressor variable. Since an intercept term will be included, a vector of 1’s is inserted into the design matrix *X*. Thus, *J*=2 indicates that, there are two columns of independent variables, first column for intercept term and second column for regressor, in the design matrix.

In this case of electrical insulating fluid data, all the three parameters including log.sigma are specified in a vector parm.names. The logposterior LP and sigma are included as monitored variables in vector mon.names. Total number of observations is specified by *N*, which is 76. Censoring is not included here. Thus, all these things are combined with object name MyData which returns the data in a list.

#### Initial values

The initial value is taken as a starting point for the estimation of a parameter. So the first two parameters, the beta parameters have been set equal to zero, and log.sigma has been set equal to log(1), which is zero.

#### Model specification

For fitting of the regression model with LaplaceApproximation, must specify a model. Thus, for failure times of electrical insulating fluid data, consider that logarithm of breakdownTime follows log-Burr distribution. In this Bayesian linear regression with an intercept and one independent variable the model is specified as

and expectation vector *μ* is an additive, linear function of vector of regression parameters, *β*, and the design matrix *X*.

Prior probabilities are specified respectively for regression coefficients, *β*, and scale parameter, *σ*,

It is obvious that all prior densities defined above are weakly informative. Thus, to specify above defined model, one must create a function called Model as:

#### Model fitting

Now, fit the above specified model using the LaplaceApproximation by assigning the object name Fit, and its results are summarized in the next section.

#### Summarizing output

The relevant summary of results of the fitted regression model using the function LaplaceApproximation, can easily be seen in these two tables. Table 5 represents the analytic result using Laplace approximation method, and Table 6 represents the simulated results using Sampling Importance Resampling method.

**Table 5:** **Posterior summary of the analytic approximation using the function** LaplaceApproximation **, which is based an asymptotic approximation theory**

Logistic model (k =1)				
**Parameter**	**Mode**	**SD**	**LB**	**UB**
Beta[1]	62.90	6.11	50.69	75.12
Beta[2]	-17.35	1.74	-20.84	-13.87
Log.sigma	-0.16	0.10	-0.35	0.04
**Weibull model (k =30)**				
**Parameter**	**Mode**	**SD**	**LB**	**UB**
Beta[1]	64.87	5.62	53.62	76.11
Beta[2]	-17.74	1.61	-20.96	-14.53
Log.sigma	0.23	0.09	0.06	0.41

**Table 6:** **Posterior summary matrices of the simulation due to sampling importance resampling algorithm using the same function**

Logistic model (k =1)							
**Parameter**	**Mean**	**SD**	**MCSE**	**ESS**	**LB**	**Median**	**UB**
Beta[1]	62.73	6.34	0.06	10000	50.08	62.84	75.23
Beta[2]	-17.30	1.81	0.02	10000	-20.88	-17.33	-13.66
Log.sigma	-0.14	0.10	0.00	10000	-0.32	-0.14	0.06
Deviance	283.20	2.46	0.02	10000	280.38	282.56	289.75
LP	-160.93	1.23	0.01	10000	-164.20	-160.61	-159.52
Sigma	0.88	0.09	0.00	10000	0.72	0.87	1.06
**Weibull model (k =30)**							
**Parameter**	**Mean**	**SD**	**MCSE**	**ESS**	**LB**	**Median**	**UB**
Beta[1]	65.18	5.86	0.06	10000	53.79	65.20	76.77
Beta[2]	-17.83	1.67	0.02	10000	-21.16	-17.83	-14.58
Log.sigma	0.25	0.09	0.00	10000	0.08	0.25	0.43
Deviance	278.35	2.40	0.02	10000	275.57	277.72	284.52
LP	-158.50	1.20	0.01	10000	-161.59	-158.19	-157.11
Sigma	1.29	0.11	0.00	10000	1.09	1.29	1.54

### Fitting with LaplacesDemon

In this section, the function LaplcesDemon is used to analyze the same data, that is, electrical insulating fluid failure times data. This function maximizes the logarithm of un-normalized joint posterior density with MCMC algorithms, and provides samples of the marginal posterior distributions, deviance and other monitored variables.

#### Model fitting

For fitting the same model with the function LaplacesDemon by assigning the object name FitDemon, the R codes are as follows. Its summary of results are printed with the function print.

#### Summarizing output

**Figure 4:**
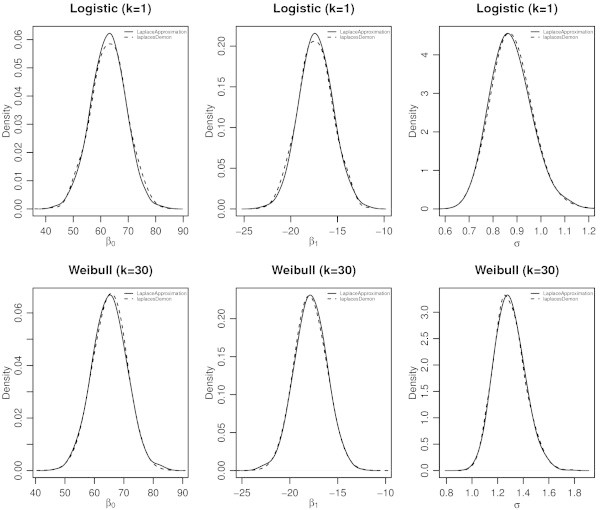
**Plot of posterior densities of the parameters** ***β***_**0**_ **,** ***β***_**1**_ **and** ***σ*** **of log-Burr model with different values of shape parameters** ***k*** **using the functions** LaplaceApproximation **and** LaplacesDemon **.**

The function LaplacesDemon for this regression model, simulates the data from the posterior density with Random-Walk-Metropolis algorithm, and summaries of results are reported in the following tables. Table 7 represents the posterior summary of all samples, and Table 8 represents the posterior summary of stationary samples. The graphical summaries of the results can also be seen in Figure 4.

**Table 7:** **Posterior summaries of simulation due to all samples using the function** LaplacesDemon

Logistic model (k =1)							
**Parameter**	**Mean**	**SD**	**MCSE**	**ESS**	**LB**	**Median**	**UB**
Beta[1]	64.45	5.90	0.32	32.69	51.07	66.30	74.19
Beta[2]	-17.80	1.68	0.09	32.38	-20.56	-18.32	-14.00
Log.sigma	-0.14	0.09	0.00	506.99	-0.31	-0.14	0.05
Deviance	283.17	2.45	0.11	518.19	280.37	282.62	288.83
LP	-160.91	1.23	0.06	518.19	-163.74	-160.64	-159.51
Sigma	0.87	0.08	0.00	508.50	0.73	0.87	1.05
**Weibull model (k =30)**							
**Parameter**	**Mean**	**SD**	**MCSE**	**ESS**	**LB**	**Median**	**UB**
Beta[1]	65.60	5.40	0.14	556.65	54.55	66.22	75.84
Beta[2]	-17.95	1.54	0.04	557.46	-20.87	-18.13	-14.77
Log.sigma	0.25	0.09	0.00	1652.97	0.08	0.25	0.44
Deviance	278.33	2.35	0.06	2000.00	275.61	277.75	284.22
LP	-158.50	1.17	0.03	2000.00	-161.44	-158.20	-157.13
Sigma	1.29	0.12	0.00	1656.80	1.08	1.29	1.55

**Table 8:** **Posterior summaries of simulation due to stationary samples using the same function**

Logistic model (k =1)							
**Parameter**	**Mean**	**SD**	**MCSE**	**ESS**	**LB**	**Median**	**UB**
Beta[1]	62.98	6.34	0.29	420.00	50.54	62.82	75.45
Beta[2]	-17.38	1.81	0.08	420.00	-20.88	-17.35	-13.85
Log.sigma	-0.13	0.09	0.00	420.00	-0.31	-0.13	0.05
Deviance	283.20	2.59	0.14	364.17	280.33	282.65	289.18
LP	-160.93	1.30	0.06	364.16	-163.92	-160.65	-159.49
Sigma	0.88	0.08	0.00	420.00	0.73	0.87	1.05
**Weibull model (k =30)**							
**Parameter**	**Mean**	**SD**	**MCSE**	**ESS**	**LB**	**Median**	**UB**
Beta[1]	65.24	5.56	0.13	1586.43	54.29	65.26	76.01
Beta[2]	-17.85	1.59	0.04	1586.97	-20.93	-17.86	-14.70
Log.sigma	0.25	0.09	0.00	1430.58	0.08	0.25	0.44
Deviance	278.37	2.37	0.06	1800.00	275.60	277.78	284.39
LP	-158.52	1.18	0.03	1800.00	-161.52	-158.22	-157.13
Sigma	1.29	0.12	0.00	1427.11	1.08	1.29	1.55

## Discussion and conclusions

In this article, Bayesian approach is applied to model the real reliability data. The generalized log-Burr distribution is used as a Bayesian model to fit the data, and for the analysis. Two important techniques, that is, asymptotic approximation and simulation method are implemented using the functions of ‘LaplacesDemon’ package of R. This package facilitates high-dimensional Bayesian inference, posing as its own intellect that is capable of impressive analysis, which is written entirely in R environment and has a remarkable provision for user defined probability model. The main body of the manuscript contains the complete description of R code both for intercept and regression models of log-Burr distribution. The function LaplaceApproximation approximates the results asymptotically and simulation is made by the function LaplacesDemon. Results of these two methods are very close to each other for different values of shape parameter *k* of log-Burr distribution. The excellency of these approximations seem clear in the plots of posterior densities. It is evident from the summaries of results that the Bayesian approach based on weakly informative priors is simpler to implement than the classical approach. The wealth of information provided in these numeric and graphic summaries are not possible in classical framework (e.g., Lawless 2003). Thus, it is very difficult to analyze these types of data by classical method, whereas it is quite simple in Bayesian paradigm using tools like R.
